# Ongoing Phenotypic and Genomic Changes in Experimental Coevolution of RNA Bacteriophage Qβ and *Escherichia coli*

**DOI:** 10.1371/journal.pgen.1002188

**Published:** 2011-08-04

**Authors:** Akiko Kashiwagi, Tetsuya Yomo

**Affiliations:** 1 Faculty of Agriculture and Life Science, Hirosaki University, Hirosaki, Aomori, Japan; 2 Graduate School of Information Science and Technology, Osaka University, Osaka, Japan; 3 Graduate School of Frontier Biosciences, Osaka University, Osaka, Japan; 4 Dynamical Micro-Scale Reaction Environment Project, Exploratory Research for Advanced Technology, Japan Science and Technology Agency, Osaka, Japan; Universidad de Sevilla, Spain

## Abstract

According to the Red Queen hypothesis or arms race dynamics, coevolution drives continuous adaptation and counter-adaptation. Experimental models under simplified environments consisting of bacteria and bacteriophages have been used to analyze the ongoing process of coevolution, but the analysis of both parasites and their hosts in ongoing adaptation and counter-adaptation remained to be performed at the levels of population dynamics and molecular evolution to understand how the phenotypes and genotypes of coevolving parasite–host pairs change through the arms race. Copropagation experiments with *Escherichia coli* and the lytic RNA bacteriophage Qβ in a spatially unstructured environment revealed coexistence for 54 days (equivalent to 163–165 replication generations of Qβ) and fitness analysis indicated that they were in an arms race. *E. coli* first adapted by developing partial resistance to infection and later increasing specific growth rate. The phage counter-adapted by improving release efficiency with a change in host specificity and decrease in virulence. Whole-genome analysis indicated that the phage accumulated 7.5 mutations, mainly in the A2 gene, 3.4-fold faster than in Qβ propagated alone. *E. coli* showed fixation of two mutations (in *traQ* and *csdA*) faster than in sole *E. coli* experimental evolution. These observations suggest that the virus and its host can coexist in an evolutionary arms race, despite a difference in genome mutability (*i.e.*, mutations per genome per replication) of approximately one to three orders of magnitude.

## Author Summary

To examine the ongoing changes driven by host–parasite interactions, we have constructed a coevolution model consisting of *Escherichia coli* and the lytic RNA bacteriophage Qβ (Qβ) in a spatially unstructured environment. In coevolution through 54 daily copropagations of the parasite and its host, *E. coli* first evolved partial resistance to infection and later accelerated its specific growth rate, while the phage counter-adapted by improving release efficiency with a change in host specificity and a decrease in virulence. Whole-genome analysis of *E. coli* and Qβ revealed accelerated molecular evolution in comparison with Qβ propagation in this study and *E. coli* sole passage reported previously. The results of the present study indicated that, despite the large difference in mutability of their genomes (approximately one to three orders of magnitude difference), a host with larger genome size (4.6 Mbp) and a lower spontaneous mutation rate (5.4×10^−10^ per bp per replication) and a parasite with a smaller genome size (4,217 bases) and a higher mutation rate (1.5×10^−3^ to 1.5×10^−5^ per base per replication) were capable of changing their phenotypes to coexist in an arms race.

## Introduction

Host–parasite coevolution has been a topic of intense research interest in various fields from basic science of molecular evolution to agricultural and medical applications [1]–[5]. According to the Red Queen hypothesis or arms race dynamics, coevolution leads to complex but continuous change, adaptation, and counter-adaptation of the phenotypes of interacting organisms [2], [6], [7]. Futuyma and Slatkin suggested that investigation of coevolution could raise and help provide answers to many questions regarding the history of evolution, *e.g.*, whether parasites tend toward specialization or toward benign or even mutualistic relationships with their hosts [8].

There have been many previous observational and theoretical studies on natural host–parasite dynamics. With regard to the relationships between bacteria and phages, Rodríguez-Valera *et al.* proposed the constant-diversity dynamics model in which the diversity of prokaryotic populations is maintained by phage predation [9]. Moreover, an observational study supported the model by analyzing the dynamics of bacteria and phages in four aquatic environments using a metagenomics method and showed that microbial strains and viral genotypes changed rapidly [10]. In addition, experimental models in simplified environments have been employed to analyze the ongoing process of coevolution. Various pairwise combinations of bacteria and phages and one with *Caenorhabditis elegans* and bacteria have been subjected to long-term laboratory cultivation [11]–[15]. These studies indicated that coevolution proceeded on a laboratory time scale [11]–[14], accelerated molecular evolution of parasites [16], [17], and broadened the host range of parasites [14]. However, the changes in genetic information and phenotype of parasites and their hosts through coevolution remain to be elucidated, and the changes in host specificity and virulence of the parasites through the arms race have not been determined in sufficient detail because ongoing adaptation and counter-adaptation in simplified experimental model systems have not been analyzed at the levels of population dynamics and molecular evolution.

To examine the ongoing changes driven by host–parasite interactions, we have constructed a coevolution model consisting of *Escherichia coli* and the lytic RNA bacteriophage Qβ (Qβ) in a spatially unstructured environment. Qβ is a simple RNA bacteriophage that infects and lyses *E. coli* cells, taking about 1 h for its burst without escaping into a lysogenic state. It has a single-stranded RNA genome of 4,217 bases encoding four genes for A2, A1 (read-through), coat protein, and RNA replicase β subunit [18]. Due to a high misinsertion rate and lack of a proofreading mechanism, ribovirus RNA replicase (including that of Qβ) has a high mutation rate [18]–[22], which allows us to monitor the evolutionary changes on a laboratory time scale.

Here, we report that in coevolution through 54 daily copropagations of the parasite and its host, *E. coli* first evolved partial resistance to infection and later showed acceleration of its specific growth rate, while the phage counter-adapted by improving release efficiency with a change in host specificity and a decrease in virulence. Fitness analysis indicated that these phenotypic changes occurred within an arms race, *i.e.*, accompanied with a monotonic fitness increase of either the parasite or its host. Whole-genome analysis indicated that the phage accumulated 7.5 mutations mainly in the A2 gene 3.4-fold faster than in Qβ propagation evolution where the phage was transferred daily to freshly prepared *E. coli* cultures, while *E. coli* showed fixation of two mutations (in *traQ* and *csdA*) faster than in sole *E. coli* experimental evolution. The results indicated ongoing adaptation and counter-adaptation through a host–parasite arms race.

## Results

### Experimental evolution system

**Figure 1:**
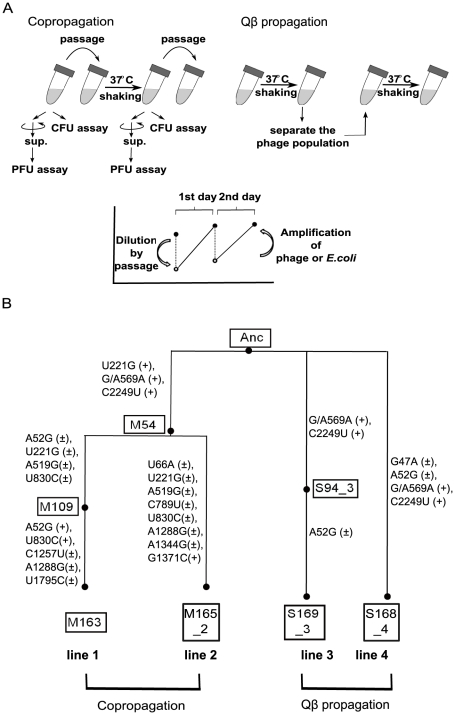
Model evolution system. (A) In the copropagation regime, cultures including *E. coli* and Qβ were passaged into fresh medium every day. In the Qβ propagation regime, only Qβ was isolated and used to infect fresh growing Anc(C). The values of PFU/ml or CFU/ml of cultures incubated for about 24 h (copropagation regime) or about 6 h (Qβ propagation regime) were determined. The concentration of Qβ and/or *E. coli* decreased by dilution in passage and increased by amplification or growth. (B) Phylogeny and nomenclature of the experimental lineages used in this study. Copropagation was conducted in two lines designated as lines 1 and line 2. Qβ propagation was conducted independently in two lines: line 3 and line 4. The ancestral organisms were designated as Anc. The M and S represent the sample of copropagation and Qβ propagation lines, respectively. The numbers in the boxes represent the replication generation numbers of Qβ, and the numbers after the underbar represent the lineage line number. The mutations observed in the Qβ genome in both propagation experiments are shown in the order of the sequence of the ancestral Qβ genome, position on the genome, and the sequence of the evolved genome. The + and ± in parentheses represent monomorphic and polymorphic sequences, respectively. Position 569 of Anc was heterogeneous with G and A, although the Anc Qβ population was derived from cloned cDNA.

Evolution experiments were carried out with copropagation of *E. coli* and Qβ and with propagation of Qβ only (Figure 1A). In the copropagation experiment, the ancestral *E. coli* strain HL2 (Anc(C)) and Qβ derived from cloned Qβ cDNA [23] (Anc(P)) were mixed, cultivated, and diluted so that the next daily culture was initiated at approximately 1×10^7^ *E. coli* cells/ml. We calculated the replication generations of Qβ genome as the cumulative generations of each passage, (N_final_/N_initial_) = 2^g^, where N_final_ and N_initial_ represent final and initial free phage density of each passage in plaque forming units (PFU/ml), respectively, and g represents replication generation. We also calculated *E. coli* cell generations as the cumulative generations of each passage, (N_f_/N_i_) = 2^n^, where N_f_ and N_i_ represent the final and initial colony forming units (CFU/ml) of each passage, respectively, and n represents cell generation. In the very early phase of the copropagation experiment, the cell generation was underestimated due to cell lysis by infection. The copropagation experimental population was divided into two on day 18, equivalent to 59 replication generations and 62 cell generations. Culture was continued to a total of 54 days (lines 1 and 2), equivalent to 163 replication generations and 163 cell generations for line 1, and 165 replication generations and 164 cell generations for line 2 (Figure 1B). Two Qβ propagation experiments, lines 3 and 4, were conducted in parallel for 18 days, equivalent to 169 and 168 replication generations where the phage population was separated daily by centrifugation from the host and transferred into fresh logarithmic cultures of the host Anc(C) (Figure 1B).

**Figure 2:**
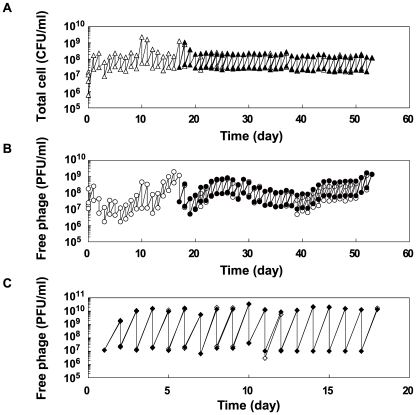
Population dynamics in the copropagation or Qβ propagation experiments. (A) Population dynamics of *E. coli* density (CFU/ml) in the copropagation experiments. Mixed cultures were divided into 2 lines on the 18^th^ day, equivalent to 59 replication generations, and propagated independently for a further 36 days (line 1 and line 2). On the 10^th^ and 17^th^ days, we restarted the copropagation experiment from stock cultures stored at −80°C. The open and closed triangles indicate the cell density of line 1 and line 2, respectively. (B) Population dynamics of free Qβ density (PFU/ml) in copropagation experiments. Open and filled circles indicate the free phage density of line 1 and line 2, respectively. (C) Population dynamics of free Qβ density (PFU/ml) in the Qβ propagation experiment. The open and filled diamonds indicate the free phage density of line 3 and line 4, respectively.

The population dynamics of the copropagation experiment demonstrated the coexistence of *E. coli* and Qβ (Figure 2), although Qβ is lytic and has no lysogenic state. The daily Qβ population density fluctuated over the course of the copropagation experiment, while the *E. coli* population density was stable probably due to the constant initial density of the host at each daily coculture. The degree of phage amplification in the copropagation experiment (2–20-fold per single coculture) was substantially lower than that in the Qβ propagation experiment (approximately 1,000-fold), even though the initial multiplicity of infection (MOI) in each passage was approximately 0.5 (approximately 10^7^ phages/ml over 2×10^7^ *E. coli* cells/ml) for the Qβ propagation experiment and was not higher than that for the copropagation experiment (Figure 2B and 2C). These observations suggested that the biotic environment for phage amplification, *i.e.*, the cellular state of the host *E. coli*, changed during the copropagation experiment.

### Fitness analysis of the parasites and hosts evolved in the copropagation experiment

Cross-cocultures were conducted to determine the changes in fitness of *E. coli* and Qβ in the copropagation experiment. Four hosts (Anc(C), M54(C), M163(C), and M165_2(C)), and the four corresponding phages (Anc(P), M54(P), M163(P), and M165_2(P)) at the 1^st^, 54^th^, 163^rd^, and 165^th^ replication generations in the copropagation experiments of lines 1 and 2 were cocultured to measure fitness in each pairwise combination. Here, the fitness of *E. coli* is defined as the ratio of the initial to the stationary optical density at 600 nm (OD_600_), while the fitness of the phage is the ratio of the initial to the stationary free phage density (PFU/ml) (Table 1, Table 2 and Figure S1).

**Table 1:** Fitness of ancestral and evolved *E. coli*.

	Qß populations				
*E. coli* populations	Anc(P)	M54(P)	M163(P)	M165_2(P)	S94_3(P)
**Anc(C)**	0.750.73	0.970.95	1.011.01	1.091.08	0.700.62
**M54(C)**	1.071.09	1.071.06	1.071.08	1.121.06	NA
**M163(C)**	1.161.15	1.161.16	1.161.15	NA	NA
**M165_2(C)**	1.151.16	1.161.16	NA	1.181.17	NA

Each experiment was conducted in duplicate and the two values are shown. The fitness of *E. coli* was calculated as (Fitness) = Log_10_ (OD_600_7h_/0.03), where OD_600_7h_ and OD_600_ = 0.03 are the OD_600_ values at 7 h after infection and initial OD_600_ of the fitness assay experiment, respectively. The growth curves are shown in Figure S1. NA, not available.

**Table 2:** Fitness of ancestral and evolved Qβ.

	*E. coli* populations			
Qß populations	Anc(C)	M54(C)	M163(C)	M165_2(C)
**Anc(P)**	4.384.20	0.410.41	0.400.35	0.340.21
**M54(P)**	4.174.11	0.880.79	0.890.85	0.680.59
**M163(P)**	3.543.46	0.930.76	1.120.98	NA
**M165_2(P)**	3.743.43	0.480.32	NA	0.830.80
**S94_3(P)**	4.884.76	NA	NA	NA

Each experiment was conducted in duplicate and the two values are shown. The fitness of Qβ was calculated as (Fitness) = Log_10_ (PFU/ml__max_/PFU/ml__0h_), where PFU/ml__max_ is PFU/ml of free phage in which we adopted the higher value between the values of 7 h or 22–29.5 h after infection, and PFU/ml__0h_ is PFU/ml of free phage just after infection. The growth curves are shown in Figure S1. NA, not available.

The host *E. coli* evolved partial resistance along with its increase in fitness (Table 1). The evolved hosts M54(C), M163(C), and M165_2(C) showed phage amplification ratios two to three orders of magnitude lower than Anc(C), regardless of whether the ancestral or evolved phage was used (host: Anc(C), M54(C), M163(C), and M165_2(C), parasite: Anc(P), M54(P), M163(P), and M165_2(P): one-way ANOVA *F_3,24_* = 213, *P*<0.01; *post hoc* Tukey–Kramer test, *P*<0.01; Table 2). The resistance was only partial, allowing phage amplification of only approximately one order of magnitude. On the other hand, the host *E. coli* infected with Anc(P) gradually showed an increase in amplification ratio along with host evolution (one-way ANOVA *F_2,3_* = 469, *P*<0.01; *post hoc* Tukey test detected significant differences between all combinations: Anc(C) *vs.* M54(C) and Anc(C) *vs.* M163(C), *P*<0.01; M54(C) *vs.* M163(C), *P*<0.05; Table 1). The growth curve of M163(C) inoculated with the phages became similar to that of the uninfected host (Figure S1A, third from left), suggesting that the host population evolved, increasing its fitness, to become almost oblivious to the phages.

Despite the development of partial resistance by the host, the phage also increased its fitness through changes in host specificity (Table 2). The evolved phage M54(P) and M163(P) showed higher fitness on the evolved host M54(C) with partial resistance than Anc(P) on the same host (one-way ANOVA *F_2,3_* = 19.7, *P*<0.05; *post hoc* Tukey test, *P*<0.05; Table 2). In line 1 and line 2, the most evolved Qβ, M163(P) or M165_2(P) showed the highest fitness on corresponding *E. coli*, M163(C) or M165_2(C), respectively. Briefly, there was a significant difference in fitness among the host–parasite combinations (host: M163(C) or M165_2(C), parasite: Anc(P), M54(P), M163(P), or M165_2(P), one-way ANOVA *F_2,3_* = 60.8, *P*<0.01; *post hoc* Tukey test, *P*<0.05 for M163(C); *F_2,3_* = 37, *P*<0.01; *post hoc* Tukey test, *P*<0.05 for M165_2(C); Table 2). The phage evolved through natural selection to show greater amplification on the corresponding host, although the amplification ratio itself decreased from approximately 10^4^ to 10^1^.

**Figure 3:**
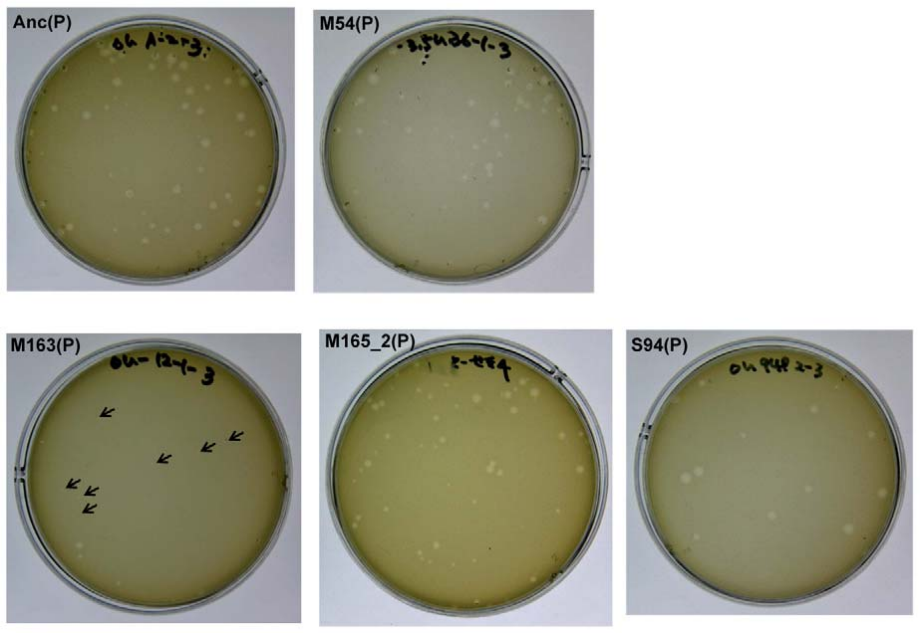
Differences in plaque size between Anc(P) and evolved Qβ. Plaque size was analyzed using A/λ as an indicator strain on LB agar medium. Most of the plaques of Anc(P) were large but some small plaques were also found. Both large and small plaques reproduced heterogeneity in plaque size (data not shown). In the copropagation regime, the plaque size decreased with number of generations, and plaques of M163(P) became quite small (indicated with arrows), while the plaques of M165_2(P) were still heterogeneous. In the Qβ propagation regime, the plaque size of S94_3(P) became uniformly large.

The phage, while responding adaptively to the evolutionary changes of its host, showed a decrease in amplification ratio on the ancestral host strain, leading to a decrease in virulence. The amplification ratios of the phage on the host Anc(C) gradually decreased over the course of the copropagation experiment (host: Anc(C), parasite: Anc(P), M54(P), M163(P), and M165_2(P), one-way ANOVA *F_3,4_* = 17.4, *P*<0.01; *post hoc* Tukey test, *P*<0.05; Table 2). A decline in phage amplification was also observed as a reduction in plaque size (Figure 3). Consequently, the evolved phage showed less cell killing effect against the ancestral strain Anc(C), resulting in better growth of the ancestral bacterial strain (host: Anc(C), parasite: Anc(P), M54(P), M163(P), and M165_2(P), one-way ANOVA, *F_3,4_* = 340, *P*<0.01; *post hoc* Tukey test, *P*<0.01; Table 1 and Figure S1A, left), *i.e.*, the phage showed a decrease in virulence. In addition, the phage evolved in the Qβ propagation experiment (S94_3(P)) showed the greatest amplification ratio (host: Anc(C), parasite: Anc(P), M54(P), M163(P), M165_2(P), and S94_3(P), one-way ANOVA *F_4,5_* = 37.8, *P*<0.01; *post hoc* Tukey test, *P*<0.05; Table 2 and Figure S1B, left) and similar virulence against Anc(C) with Anc(P) (host: Anc(C), parasite: Anc(P) and S94_3(P), Welch's *t* test, *t* = 12.7, *P* = 0.45; Table 1 and Figure S1A, left). These results suggest that the decrease in virulence was not due to simple degeneration through the long-term passage experiment, but was probably at the expense of increasing the fitness of the phage in the arms race with its host.

### Mechanism of fitness improvement

To examine how *E. coli* and Qβ improved their fitness during coevolution, free phages, infected *E. coli* cells, and total *E. coli* cells from the copropagation of line 1 were monitored hourly by determining the numbers of PFUs in the supernatant and pellet after centrifugation and CFU, respectively (see Materials and Methods).

**Figure 4:**
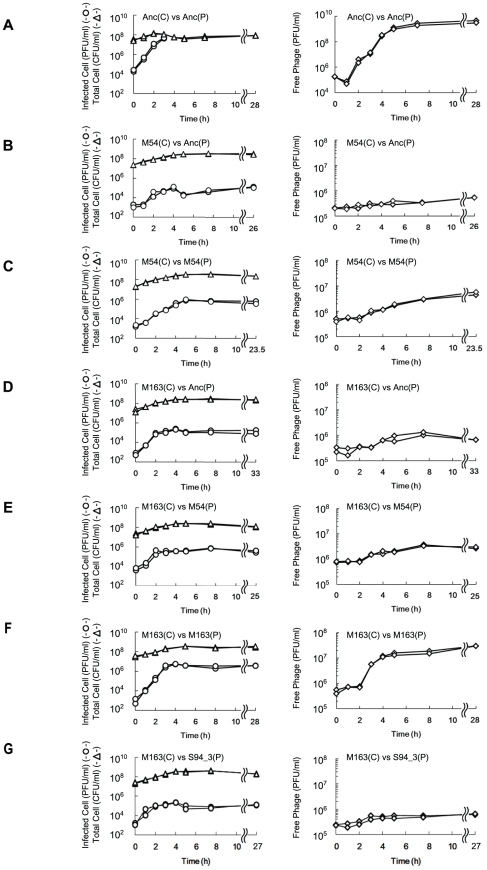
Time course of changes in density of free phage, infected *E. coli*, and total *E. coli*. The time course of changes in free phage density (PFU/ml) (◊), infected cell density (PFU/ml) (○) and total cell density (CFU/ml) (Δ) for (A) Anc(C) infected with Anc(P), (B) M54(C) infected with Anc(P), (C) M54(C) infected with M54(P), (D) M163(C) infected with Anc(P), (E) M163(C) infected with M54(P), (F) M163(C) infected with M163(P), and (G) M163(C) infected with S94_3(P). Experiments were performed in duplicate. The rates of infected cell fitting straight lines to semi-logarithmic plots of infected cell density for (A) to (G) were 2.62 h^−1^ (r^2^ = 0.98), 1.21 h^−1^ (r^2^ = 0.89), 1.26 h^−1^ (r^2^ = 0.98), 2.40 h^−1^ (r^2^ = 0.98), 2.80 h^−1^ (r^2^ = 0.92), 2.72 h^−1^ (r^2^ = 0.99), and 2.13 h^−1^ (r^2^ = 0.91), respectively. The amplification rates of free phage fitting straight lines to semi-logarithmic plots of free phage density for (A) to (G) were 2.43 h^−1^ (r^2^ = 0.97), 0.11 h^−1^ (r^2^ = 0.43), 0.31 h^−1^ (r^2^ = 0.93), 0.25 h^−1^ (r^2^ = 0.79), 0.24 h^−1^ (r^2^ = 0.89), 1.38 h^−1^ (r^2^ = 0.93), and 0.27 h^−1^ (r^2^ = 0.74), respectively.

**Figure 5:**
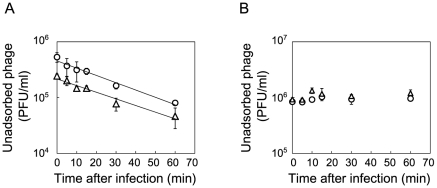
Adsorption rate constants of Anc(P) and M163(P). The concentration of unadsorbed phage was measured as PFU/ml to estimate the Qβ phage adsorption rate to Anc(C) or M163(C). (A) Anc(P) (○) and M163(P) (Δ) adsorption to Anc(C) were analyzed (*n* = 3). Values are shown as means ± SD. The rate constants for adsorption of both Qβ to Anc(C) were the same (1.4×10^−9^ ml/cells/min) estimated from the slopes of the regression curves. (B) Anc(P) (○) and M163(P) (Δ) adsorption to M163(C) were analyzed (*n* = 3). Values are shown as means ± SD.

**Figure 6:**
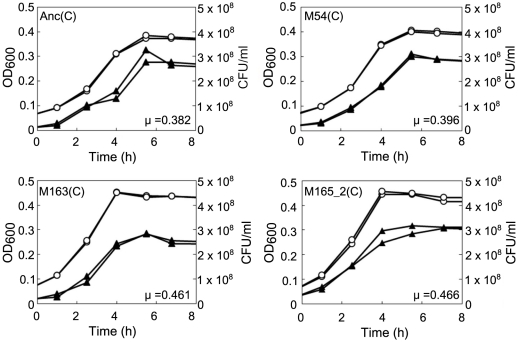
Growth curves of ancestral and evolved *E. coli*. The growth curves of Anc(C), M54(C), M163(C), and M165_2(C) without phage infection were plotted based on OD_600_ and CFU/ml. Each experiment was conducted in duplicate. The open circles and closed triangles represent the OD_600_ and CFU/ml, respectively. The specific growth rate (μ) calculated from the semi-logarithmic plots based on OD_600_ of Anc(C), M54(C), M163(C), and M165_2(C) were 0.382 h^−1^ (r^2^ = 0.998), 0.396 h^−1^ (r^2^ = 0.996), 0.461 h^−1^ (r^2^ = 0.998), and 0.466 h^−1^ (r^2^ = 0.996), respectively. The times to reach stationary phase based on OD_600_ of Anc(C), M54(C), M163(C), and M165_2(C) were 4.60 and 4.57 h, 4.47 and 4.44 h, 3.86 and 3.83 h, and 3.88 and 3.82 h, respectively.

*E. coli* was found to first evolve partial resistance to Qβ, which was followed by a later increase in the specific growth rate. After 3 hours of incubation with Anc(P), almost all of the ancestral host Anc(C) cells were infected, while infection ratios of the evolved hosts M54(C) and M163(C) were only 0.03% and 0.08%, respectively (Figure 4A, 4B, and 4D, left). The observed partial resistance was likely due to a very low adsorption rate of *E. coli* cells to the phage (Figure 5). As most of the evolved host cells remained uninfected, they were able to proliferate, while the ancestral cells could not. In addition, the uninfected cells of the most evolved hosts, M163(C) and M165_2(C) for lines 1 and 2, respectively, showed higher specific growth rates than those of M54(C) (see legend of Figure 6 for specific growth rates, ANCOVA, *F_2,24_* = 18.0, *P*<0.001; *post hoc* Tukey test, *P*<0.001). Although the OD_600_/CFU seemed to have changed over the copropagation experiments (Figure 6), the same conclusion was obtained using specific growth rates based on CFU values (data not shown). Briefly, M54(C) eliminated Anc(C) from the population by developing partial resistance to phage infection, and M163(C) and M165_2(C) finally took over the population due to acceleration of specific growth rate.

The phages evolved to show increased release efficiency, *i.e.*, the number of phages released from a single infected cell per unit time. As the phage evolved, the speed of free phage amplification for either M54(C) or M163(C) increased (the amplification rates of free phage density of Anc(P) and M54(P) on M54(C) were 0.11 h^−1^ (r^2^ = 0.43) and 0.31 h^−1^ (r^2^ = 0.93), respectively, two-tailed *t* test *t* = 3.24, *P*<0.01; Figure 4B and 4C, right, and those of Anc(P), M54(P), and M163(P) on M163(C) were 0.25 h^−1^ (r^2^ = 0.79), 0.24 h^−1^ (r^2^ = 0.89), and 1.38 h^−1^ (r^2^ = 0.93), respectively, ANCOVA; *F_2,18_* = 35.3 *P*<0.01; *post hoc* Tukey test, *P*<0.01; Figure 4D, 4E, and 4F, right), while the infection efficiency, *i.e.*, the rate of increase in infected cells, did not change significantly for the same hosts (the rates of increase in infected cells of M54(C) infected with Anc(P) or M54(P) were 1.21 h^−1^ (r^2^ = 0.89) and 1.26 h^−1^ (r^2^ = 0.98), respectively, two-tailed *t* test, *t* = 0.39, *P* = 0.70; Figure 4B and 4C, left, and those of M163(C) infected with Anc(P), M54(P), or M163(P) were 2.40 h^−1^ (r^2^  =  0.98), 2.80 h^−1^ (r^2^ = 0.92), and 2.72 h^−1^ (r^2^ = 0.99), respectively, ANCOVA, *F_2,12_* = 0.95, *P* = 0.41; Figure 4D, 4E, and 4F, left). The acceleration of free phage amplification rate could be attributed to either an increase in burst frequency per unit time or burst size. There was no significant difference in burst size between M163(P) and Anc(P) on M163(C) determined by the method of analysis of burst sizes in single cell [24] (data not shown). Therefore, the phage seems to have evolved to burst more frequently from infected cells per unit time. It is noteworthy that the most evolved phage, M163(P), inoculated onto M163(C) showed a marked increase in number of free phage at 3 h, probably leading to further infection of surrounding uninfected hosts and an increase in proportion of infected cells beyond the inoculated free phage concentration (4.8×10^5^ PFU/ml) (Figure 4F). Other phages stopped increasing the number of infected cells at around the inoculated free phage concentration (Figure 4D, 4E, and 4G).

### Molecular evolution of host and parasite

**Figure 7:**
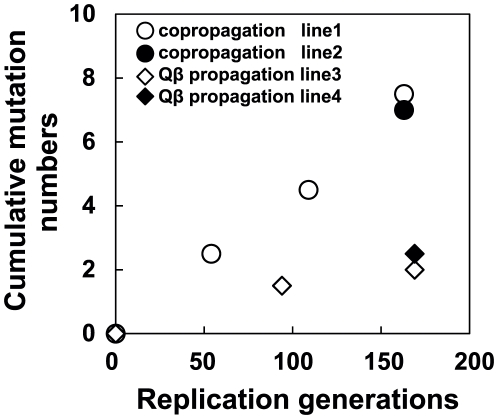
Time course of changes in cumulative mutations in the Qβ genome. The cumulative mutations in line 1 (•), line 2 (○), line 3 (▴), and line 4 (Δ) are shown. Cumulative mutation numbers were calculated as described in the legend of Table 3.

We performed whole genome sequence analyzes of all of the Qβ populations indicated in Figure 1B to determine how molecular evolution of the phage proceeded in response to the adaptation of the hosts. First, mutations were shown to be accumulated in a biased manner in the A2 gene, which encodes a multifunctional protein related to infection and cell lysis (Figure 1B and Table 3). The A2 gene, accounting for 30% of the whole genome, accumulated 65.5% of all mutations, and this bias was shown to be statistically significant (*P*<0.05, two-tailed binomial test). A similar substantial accumulation of mutations in genes related to host infection was demonstrated previously in an evolution experiment using the DNA bacteriophage Φ2 [16]. The mutation fixation rate in phage was higher in the copropagation experiment (1.0×10^−5^±6.0×10^−7^ per base per generation) than that in the Qβ propagation experiment (3.2×10^−6^±5.1×10^−7^ per base per generation) (two-tailed Welch's *t* test *t* = 4.3, *P*<0.01), suggesting that the phage showed accelerated molecular evolution through coevolution with its host (Figure 7).

**Table 3:** Nucleotide sequences in the ancestral and evolved phage genomes.

Phage Population									S/N	Gene	Genome position	Nucleotide (Anc)	Nucleotide (Evolved)	Gene position	Codon change	Amino acid change
NCBI (AY099114)	Anc	M54	M109	M163	M165_2	S94_3	S169_3	S168_4								
								**±**	**–**	**UTR**	**47**	**G**	**A**	**–**	**–**	**–**
			**±**	**+**			**±**	**±**	**–**		**52**	**A**	**G**	**–**	**–**	**–**
					**±**				**S**	***A2***	**66**	**U**	**A**	**6(2)**	**CCU→CCA**	**–**
		**+**	**±**		**±**				**N**		**221**	**U**	**G**	**161(54)**	**CUA→CGA**	**Leu→Arg**
			**±**	**±**	**±**				**S**		**519**	**A**	**G**	**459(153)**	**GAA→GAG**	**–**
**+**	**±**	**+**	**+**	**+**	**+**	**+**	**+**	**+**	**N**		**569**	**G/A**	**A**	**509(170)**	**GGG→GAG**	**Gly→Glu**
					**±**				**S**		**789**	**C**	**U**	**729(243)**	**GGC→GGU**	**–**
			**±**	**+**	**±**				**N**		**830**	**U**	**C**	**770(257)**	**GUU→GCU**	**Val→Ala**
**+**				**±**					**S**		**1,257**	**C**	**U**	**1,197(399)**	**ACC→ACU**	**–**
				**±**	**±**				**N**		**1,288**	**A**	**G**	**1,228(410)**	**AGU→GGU**	**Ser→Gly**
					**±**				**S**	***A1 coat***	**1,344**	**A**	**G**	**1(1)**	**AUG→GUG**	**Met→Met** *
					**+**				**N**		**1,371**	**G**	**C**	**28(10)**	**GGU→CGU**	**Gly→Arg**
				**±**					**N**	***A1***	**1,795**	**U**	**C**	**452(151)**	**AUU→ACU**	**Ile→Thr**
**+**		**+**	**+**	**+**	**+**	**+**	**+**	**+**	**S**		**2,249**	**C**	**U**	**906(302)**	**AGC→AGU**	**–**

Blank, “±,” and “+” in the phage population column indicate that the whole population showed the same sequence as Anc, heterogeneous with Anc and evolved sequence, and evolved sequence, respectively. S and N in the S/N column represent synonymous and non-synonymous mutations, respectively. UTR, untranslated region. Genome positions were counted from the G of the 5′ terminus of the Qβ genome as position 1. Gene position was counted from the A of the start codon (AUG) of each gene as position 1. The numbers in parentheses represent the amino acid positions of each protein. Position 569 of Anc was heterogeneous with G and A, although the Anc Qβ population was derived from cloned cDNA.

*This mutation was AUG to GUG in the start codon. If this protein was functional, the GUG start codon would be used to encode Met.

To determine mutation fixation and cumulative mutation numbers in Figure 7, polymorphic sites (shown as ±) were counted as 0.5, and monomorphic sites (shown as +) were counted as 1. For position 221 in line 1, a count of 1 was given from Anc(P) to M54(P), 0.5 from M54(P) to M109(P), and 0.5 from M109(P) to M163(P). For position 221 in line 2, a count of 1 was also given from Anc(P) to M54(P) and 0.5 from M54(P) to M165_2(P).

Whole-genome analysis of *E. coli* revealed the process of molecular evolution in the host cells. We analyzed the whole genome sequence of M163(C) using an Illumina Genome Analyzer IIx (GAIIx; Illumina, San Diego, CA) and confirmed the mutations in M163(C) together with Anc(C) and M54(C) by the dideoxynucleotide chain termination sequencing method [25]. A single mutation in *traQ* (S21P) encoded on the F plasmid was detected in M54(C) and an additional mutation was detected in *csdA* (D340N) in M163(C) (Table S1, Table S2). As discussed below, the protein products from these genes may contribute to resistance to phage infection and the increase in fitness of *E. coli*.

## Discussion

### Phenotypic and genetic changes of Qβ and *E. coli*

In the copropagation experiment, *E. coli* adopted a simple strategy with only two mutations, while the phage accumulated more mutations within its small genome as counter-adaptation against the evolutionary changes in the host. The host first developed resistance to phage infection *via* a non-synonymous mutation in *traQ*. This gene encodes TraQ, the conjugal transfer pilin, which is a component of the F pilus, and is a chaperone for inserting propilin into the inner membrane. Propilin was reported to be unstable in *traQ* ^−^ cells [26], and amino acid 21 of TraQ where the mutation was detected in this study interacts with propilin [27]. F pilus assembly from membrane F-pilin requires many Tra proteins [28]. As no mutations were detected in other Tra protein genes in the copropagation experiment, the mutation on TraQ may result in a decrease in the amount of inserted propilin, leading to the partial resistance observed in this study. *E. coli* then showed further mutation in *csdA*, which encodes CsdA, an enzyme related to Fe/S biogenesis and a new sulfur transfer pathway that is related to the fitness of these cells, especially in stationary phase [29]. Therefore, this mutation could be beneficial as the host was passaged daily at the stationary phase in the evolutionary experiment.

On the other hand, the phage evolved to increase release efficiency by accumulating mutations mostly in the gene encoding the A2 protein. A2 is a multifunctional protein with roles in host cell lysis, adsorption to the F pilus of *E. coli*, RNA binding during capsid assembly, protection of the 3′ terminus, penetration into the cytoplasm of the host, and blockage of cell wall biosynthesis by inhibiting the catalytic step from UDP-GlcNAc to UDP-GlcNAc-EP catalyzed by MurA [18], [30]–[32]. Due to the cell lytic activity of A2, it is unsurprising that these mutations might have resulted in an increased burst frequency and release efficiency. In fact, the experimental lag period between infection and detection of the increase in free phage became shorter by approximately 1 hour in the cross-culture experiment (*e.g.*, 3 h for Anc(P) and 2 h for M163(P) on M163(C), Figure 4D and 4F, right, respectively). It should be noted that the uninfected M163(C) reached the stationary phase, which was not susceptible to phage infection, approximately 1 hour earlier than the other hosts (see legend of Figure 6 for the time to reach the stationary phase, one-way ANOVA, *F_2,3_* = 693.8, *P*<0.001; *post hoc* Tukey test, *P*<0.001). Thus, it is possible that the increased burst frequency of M163(P) for the earlier phage release evolved as a counter-adaptation on the evolved host M163(C) due to the shorter period available for infection. Previous experiments using DNA bacteriophages indicated that shorter latent periods were favored in the presence of a high density of highly susceptible host cells [33], [34].

It is of interest that Qβ evolved to show reduced virulence toward the ancestral host. Many studies have indicated that phages with low or moderate virulence were favored in vertical transmission or in structured environments [35]–[37], while Qβ has no lysogenic state and evolved reduced virulence in this experiment. The decrease in virulence observed in this study may have been a side effect of the increase in burst frequency. If fact, the evolved phage M163(P) with increased burst frequency on the evolved host M163(C) showed lower virulence and lower fitness on Anc(C) than Anc(P) on Anc(C) (Figure 4 and Figure S1 left), suggesting that Qβ may have co-evolved to increase the burst frequency in reducing some benefits that can be gained if the host reverts to the Anc(C)-like phenotype.

The single non-synonymous mutation at position 221 of the A2 gene found in M54(P) seems to have resulted in reduced virulence and a change in host specificity. As the non-synonymous mutation was only observed in the copropagation experiment and two others were also observed in Qβ propagation and the deposited sequence (NCBI accession no. AY099114), the mutation at 221 and/or the combinations with the mutation and two other mutations may have resulted in the decrease in virulence and the change in host specificity observed in M54(P).

### Accelerated molecular evolution rates of Qβ and *E. coli*

In coevolution between Qβ and its host, *E. coli*, the phage showed accelerated molecular evolution (Figure 7). In the Qβ propagation experiment, the molecular evolution of the phage proceeded but seemed to slow down after the 94^th^ generation. On the other hand, the phage coevolving with *E. coli* retained a 3.4-fold faster molecular evolution rate throughout the copropagation experiment. The higher evolution rate may be attributable to the changes occurring in the host *E. coli*. If the host had stopped evolving, *e.g.*, at the 54^th^ generation, the M163(P) or M165_2(P) phage would not have been fixed into the population as it had fitness similar to or less than that of M54(P), leading to deceleration of evolutionary rate. It should be noted that neutral mutations cannot be fixed in the copropagation experiment because 163 replication generations is too short for them to become fixed. The fixation of neutral mutations is known to require generations approximately as long as the effective population size (Ne) [38]. The effective population size in the copropagation experiment was roughly estimated as the bottleneck size of the population (approximately 10^3^ phages) assuming that 1% of the minimum initial 10^6^ phages infect and burst to release approximately 10^7^ phages. Thus, even synonymous mutations observed here were positively selected [38], consistent with the influence of the RNA secondary structure on Qβ genome replication reported previously [39]–[42]. Some synonymous mutations may have physiological impacts on phage growth because of genomic secondary structure; it has been reported that some synonymous mutations or mutations in intergenic regions show lethal effects in Qβ [42].

It is noteworthy that the fixation rate of the *E. coli* genome in the copropagation regime (2.6×10^−9^ per bp per generation) calculated as 2 mutations in 4.73 Mbp per 163 generations was one order of magnitude higher than that under conditions of *E. coli* sole passage, maintaining log phase at 37°C (1.7×10^−10^ per bp per generation) [43] or 20,000 generations (1.6×10^−10^ per bp per generation) (Poisson distribution, *P*<0.01) [44]. In summary, these observations indicated that molecular evolution rates of both the parasite and its host were accelerated through adaptation and counter-adaptation.

### The arms race in the evolution experiment

**Figure 8:**
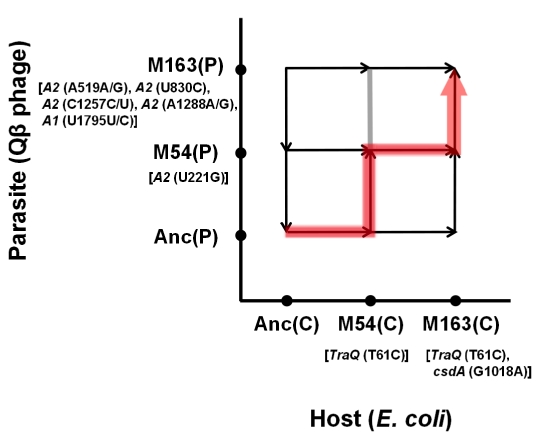
Estimation of evolutionary path. The direction of the arrowhead on each arrow indicates higher fitness. The gray line at M54(C) indicates that M54(P) and M163(P) showed almost equivalent fitness on M54(C). The red line indicates the plausible route for fixing M163(C) and M163(P) in this study. The genes in which mutations compared with the ancestor and detected only in the copropagation regime are shown in brackets, and the mutation positions in each gene for *E. coli* and in the Qβ genome are shown in parentheses.

Based on the observed fitness changes in the host *E. coli* and in the Qβ phage, we propose a plausible coevolution path to depict the arms race between Qβ and the host *E. coli*. As the order of phage fitness on the ancestral *E. coli* Anc(C) was Anc(P)>M54(P)>M163(P), the population in the coculture seemed to first take a route not in the direction of phage evolution (upward) but in the direction of host evolution (right), increasing host fitness by increasing its resistance to Qβ (Figure 8). The arrows in Figure 8 reflect the experimentally determined finesses changes (Table 1 and Table 2). Arriving around the pair position of M54(C) *vs.* Anc(P), the population could take either the upward or rightward direction, but happened to take the direction of phage evolution due to the occasional appearance of a single non-synonymous mutation at position 221 in the phage genome that was detected only under copropagation conditions (Table 3). The population of M54(P) and M54(C) could not fix a phage mutant like M163(P) with the same fitness as M54(P), but fixed the *E. coli* mutant M163(C) with fitness higher than that of M54(C). Due to the host change from M54(C) to M163(C) accompanied with an additional single non-synonymous mutation in *csdA*, the phage mutant M163(P) was fitter than M54(P) and was therefore fixed in the final population. Taken together, these findings indicated that the evolutionary path seemed to be an arms race involving adaptation of *E. coli* and counter-adaptation of the phage.

We showed that parasites, such as RNA viruses, and hosts, such as *E. coli*, have the potential to coexist even in an arms race. When a parasite encounters its host, the host may become extinct through the evolution of high parasite virulence, or the parasite may become extinct through the evolution of host resistance. However, both may also change their phenotypes by genomic mutation in a synchronized manner and thus coexist. The results of the present study indicated that a host with a larger genome size (4.6 Mbp) with a low spontaneous mutation rate (5.4×10^−10^ per bp per replication) [45] and a parasite with a smaller genome size (4,217 bases) and a higher spontaneous mutation rate (1.5×10^−3^–10^−5^ per base per replication) [18]–[22], despite the large difference in mutability of their genomes (approximately one to three orders of magnitude difference), were capable of changing their phenotypes to coexist in an arms race. Further studies linking the phenotype mutability and genome complexity will help to elucidate the dynamic host–parasite relationship.

## Materials and Methods

### Strains and media

The *E. coli* HL2 strain was used as the coculture host strain and A/λ [46] was used as an indicator strain for the titer assay. The *E. coli* HL2 strain was constructed by conjugation with DH1Δ*leuB*::(*gfpuv5*-Km^r^) [43] and HB2151 [47]. We mixed log-phase DH1Δ*leuB*::(*gfpuv5*-Km^r^) and HB2151 for 2.5 hours and screened for kanamycin-resistant clones on LB agar medium supplemented with 25 µg/ml kanamycin. F′ retention of HL2 was checked by PCR with the primers TraU_f (5′-ATGAAGCGAAGGCTGTGGCT-3′) and TraU_r (5′-GCAGCTTGAACGCCATGCGT-3′) and the ability of HL2 to amplify Qβ was confirmed. Before the evolution experiments, HL2 was grown in mM63gl (62 mM K_2_HPO_4_, 39 mM KH_2_PO_4_, 15 mM ammonium sulfate, 1.8 µM FeSO_4_·7H_2_O, 15 µM thiamine hydrochloride, 2.5 mM MgSO_4_·7H_2_O, 0.04% glucose, and 1 mM l-Leu) for several passages until the specific growth rate had become stable, and the strain with stable growth rate was used as the ancestor strain (Anc(C)). The OD_600_ of stationary-phase Anc(C) cultured in mM63gl medium was approximately 0.4 (approximately 3×10^8^ CFU/ml) because of glucose limitation. Qβ was kindly provided by Dr. Koji Tsukada (Osaka University, Japan), which was generated from Qβ genomic cDNA [23]. Qβ particles were diluted with LB medium and plaque assay was performed according to the standard method [48]. Polypropylene centrifuge tubes (15 ml, No. 430791; Corning Incorporated, Corning, NY) treated with 0.1% BSA for at least 15 minutes to prevent attachment of phages to the tube walls were used for all the experiments as culture tubes.

### Experimental evolution system

Copropagation experiment: 4.8×10^7^ cells Anc(C) and 5.1×10^7^ PFU Anc(P) were mixed and copropagation was started in a culture volume of 3 ml at 37°C with shaking at 160 rpm. Mixed cultures were divided into 2 lines on the 18^th^ day, equivalent to 59 replication generations, and propagated independently for a further 36 days (line 1 and line 2 in Figure 1B). Serial transfer was conducted by daily transfer of the cultures with cells and phages. The portion of cultures calculated based on the final OD_600_ were transferred into fresh medium with dilution to an initial OD_600_ of 0.05. Daily culture samples were divided into thirds: one for preparing −80°C frozen stocks with 15% glycerol, one for CFU determination by dilution and spreading on low divalent cation mM63gl agar medium with 0.2 mM MgSO_4_·7H_2_O, and the other for PFU analysis using the supernatant after centrifugation. Qβ propagation experiment: Two lines (line 3 and line 4 in Figure 1B) were independently propagated from Anc(P) for 18 days, equivalent to 168–169 replication generations, at 37°C with shaking at 160 rpm. Serial passages consisted of infection of a host culture, followed by about 6 h of phage growth, and extraction of the phage from the culture. Each serial passage was performed as follows: uninfected Anc(C) cultures were grown at 37°C overnight and transferred into new medium with dilution to OD_600_ of 0.03. When OD_600_ became 0.06–0.07 (approximately 1×10^7^ CFU/ml) after 2–2.5 h, cells were infected with phage to approximately 1.0–2.0×10^7^ PFU/ml from the previous passage. The cultures were grown for about 6 h. *E. coli* cells were removed by centrifugation, and the supernatant was subjected to filtration with 0.2 µm syringe filters (Minisart RC15 filters; Sartorius Stedim Biotech, Goettingen, Germany), and phage solution was stored at 4°C for infection on the next serial passage. The replication generation number of the phage population (n) was calculated as n =  ln_2_ (N_f_/N_i_), where N_i_ and N_f_ are the phage density (PFU/ml) at the initial and final time points of each passage, respectively. The initial value (N_i_) was calculated by dividing the N_f_ of the previous passage by the dilution rate.

### Purification of *E. coli* and Qβ phage from mixed cultures

The evolved *E. coli* populations (M54(C), M163M(C), and M165_2(C)) and Qβ phage populations (M54(P), 163(P), and M165_2(P)) were purified from mixed cultures to analyze the phage genome sequence and to determine their fitness. To purify the evolved *E. coli* population, cultures stocked at −80°C including evolved *E. coli* and phage were streaked on mM63gl agar medium and then passaged several times in low divalent cation medium, 0.2 mM MgSO_4_·7H_2_O mM63gl, to prevent further phage adsorption to *E. coli*. We checked the purity of evolved *E. coli* by confirming that no plaques were observed in the passaged and chloroform-treated cultures. To purify the evolved phage population, cultures stocked at −80°C including evolved *E. coli* and phage were cultured in mM63gl at 37°C with shaking at 160 rpm for 1 day and filtrated with 0.2 µm syringe filters (Minisart RC15 filters; Sartorius Stedim Biotech). These particles were used for RNA genome sequencing analysis as described below. For analysis of phage fitness, these filtrated particles and Anc(P) were dialyzed to remove carry-over glycerol from the −80°C stock using Microcon centrifugal filter devices with 10,000 nominal molecular weight limit membranes (Millipore, Billerica, MA). We confirmed that the dialysis step did not affect plaque forming ability.

### Genome sequencing of Qβ

The RNA genomes of the Qβ population noted in Figure 1B, *i.e.*, 8 kinds of genome derived from approximately 10^8^ PFU particles of the Anc(P), M54(P), M109(P), M163(P), M165_2(P), S94_3(P), S169_3(P), and S168_4(P) phage populations, were extracted using a QIAamp Viral RNA mini kit (Qiagen, Hilden, Germany) according to the manufacturer's instructions. To analyze the full-length RNA genome sequence, samples were prepared as follows. Poly(A) was added at the 3′ end using poly(A) polymerase (Applied Biosystems/Ambion, CA). cDNA was synthesized using the primer Qt (5′-CCAGTGAGCAGAGTGACGAGGACTCGAGCTCAAGCTTTTTTTTTTTTTTTTT-3′) with SuperScript™ III Reverse Transcriptase (Invitrogen, Carlsbad, CA), and then RNA was degraded with RNaseH. The first-strand cDNA was purified and poly(A) was added at the 3′ termini of the cDNA with terminal deoxynucleotidyl transferase (Roche Diagnostics, Basel, Switzerland) and dATP. The cDNA with poly(T) at the 5′ terminus and poly(A) at the 3′ terminus was purified. To obtain the 5′ end of the Qβ phage genome sequence, second-strand DNA was prepared using Qt primer and PfuUltra II Fusion HS DNA Polymerase (Stratagene, La Jolla, CA). PCR was performed separately with high fidelity DNA polymerases for the whole Qβ genome divided into 5 regions as shown in Table S3. In total, PCR products were obtained from cDNA template derived from approximately 10^6^ PFU particles. The templates, primers, and polymerase used for PCR and the primers used for sequencing are listed in Table S3. Sequencing was performed by the dideoxynucleotide chain termination method [25] on both strands, but we conducted direct sequencing in one direction using 2 sets of primers only for the 5′ end of the genome (Table S3). When a double peak appeared in the sequencing chart, positions where the height of the smaller peak was over half that of the larger peak were defined as polymorphic sites.

### Genome sequencing of *E. coli*

Genomic DNA was extracted from over 10^9^ cells of Anc(C), M54(C), and M163(C) using a DNeasy Blood & Tissue Kit (Qiagen) according to the manufacturer's instructions. The genomic DNA of M163(C) was sequenced with an Illumina GAIIx (Illumina) using 51-bp of single-read format by Hokkaido System Science Co., Ltd. (Sapporo, Hokkaido, Japan). The GAIIx produced 18,718,549 reads and 954,646 kb. All reads were aligned to the reference sequence of *E. coli* DH1 genome sequence (GenBank accession number, CP001637.1; genome size, 4,630,707 bp) and F plasmid sequence (GenBank accession number, NC_002483.1; size, 99,159 bp) using MAQ [49] guaranteed to find alignments with up to 2 mismatches in the first 24 bp of the reads and 5 mismatches in 51 bp. Mean depth was 196 dividing 927,788 kb mapped bases by 4,730 kb of total reference sequences. After mapping and consensus base calling, SNPs were filtered with the same threshold values as reported previously [49]. The alignment view was also confirmed with Mapview [50], and SNPs were also scored with the following parameters: Phred quality score ≥20, variant frequency ≥0.40, coverage sum ≥5. In addition, Tablet [51] also has the ability to align short reads to the reference sequence, thus allowing us to score sites with a deletion, insertion, and/or regions with large insertions or deletions. SNPs, insertions, and deletions were scored for the F plasmid and for the M163(C) genome, and they were confirmed by sequencing using the dideoxynucleotide chain termination method for the genomes derived from over 10^7^ cells of Anc(C) and M163(C) using the primers listed in Table S1. Two different positions observed in Anc(C) and M163(C) were sequenced for M54(C) by the dideoxynucleotide chain termination method.

### Fitness assay

The cross-coculture experiment, time course analysis after infection, and adsorption rate constant analysis were conducted according to the methods described in Text S1.

### Statistical analysis

Fitness and the time to reach the stationary phase were compared by one-way ANOVA with the *post hoc* Tukey test [52]. The virulence of Anc(P) and S94_3(P) to Anc(C) was compared with Welch's *t* test. The differences in growth rates calculated from semi-logarithmic plots of *E. coli* or phage densities were tested by two-tailed *t* test and ANCOVA with the *post hoc* Tukey test [53].

## Supporting Information

Figure S1 Growth curves of *E. coli* and Qβ in the cross-coculture experiment. Each experiment was conducted in duplicate and the results were reproducible. Filled and open symbols are from duplicate measurements. (A) The growth curves of *E. coli* infected with phages are represented. The following growth curves are shown from left to right: Anc(C) infected with Anc(P), M54(P), M163(P), M165_2(P), and S94_3(P), and without phage infection; M54(C) infected with Anc(P), M54(P), M163(P), and M165_2(P), and without phage infection; M163(C) infected with Anc(P), M54(P), and M163(P), and without phage infection; and M165_2(C) infected with Anc(P), M54(P), and M165_2(P). (B) The growth curves of free phage are also shown (from left to right): Anc(P), M54(P), M163(P), M165_2(P), and S94_3(P) amplified on Anc(C); Anc(P), M54(P), M163(P), and M165_2(P) amplified on M54(C); Anc(P), M54(P), and M163(P) amplified on M163(C); and Anc(P), M54(P), and M165_2(P) amplified on M165_2(C).
(PDF)

Table S1 Genome and F′ sequencing of *E. coli*.
(XLS)

Table S2 Deleted genes indicated by ** and *** in Table S1.
(XLS)

Table S3 List of templates, primers, and polymerase used for Qβ genome sequencing.
(XLS)

Text S1 Supporting Materials and Methods.
(DOC)
