# Supplementary figures and images for: Ongoing Phenotypic and Genomic Changes in Experimental Coevolution of RNA Bacteriophage Qβ and Escherichia coli
Source: PLoS Genet. 2011 Aug 4;7(8):e1002188. doi: 10.1371/journal.pgen.1002188 (PMC3150450; doi:10.1371/journal.pgen.1002188)

A

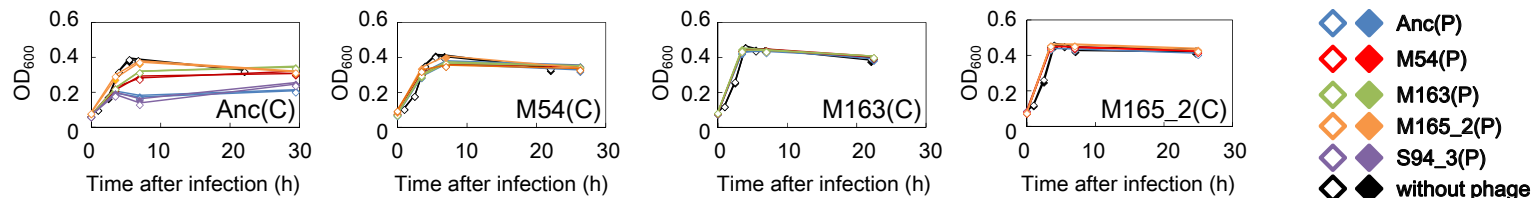

B

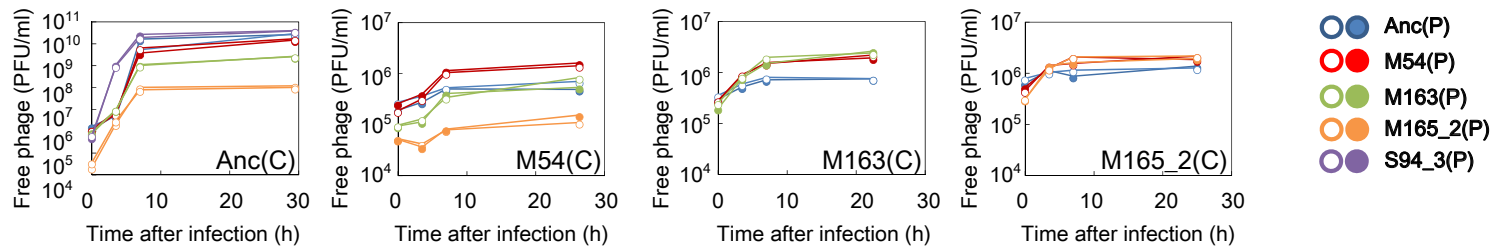

Supplement: Figure S1 — Growth curves of E. coli and Qβ in the cross-coculture experiment. Each experiment was conducted in duplicate and the results were reproducible. Filled and open symbols are from duplicate measurements. (A) The growth curves of E. coli infected with phages are represented. The following growth curves are shown from left to right: Anc(C) infected with Anc(P), M54(P), M163(P), M165_2(P), and S94_3(P), and without phage infection; M54(C) infected with Anc(P), M54(P), M163(P), and M165_2(P), and without phage infection; M163(C) infected with Anc(P), M54(P), and M163(P), and without phage infection; and M165_2(C) infected with Anc(P), M54(P), and M165_2(P). (B) The growth curves of free phage are also shown (from left to right): Anc(P), M54(P), M163(P), M165_2(P), and S94_3(P) amplified on Anc(C); Anc(P), M54(P), M163(P), and M165_2(P) amplified on M54(C); Anc(P), M54(P), and M163(P) amplified on M163(C); and Anc(P), M54(P), and M165_2(P) amplified on M165_2(C). (PDF) [file pgen.1002188.s001.pdf]
