# Supplementary material for: Ongoing Phenotypic and Genomic Changes in Experimental Coevolution of RNA Bacteriophage Qβ and Escherichia coli
Source: PLoS Genet. 2011 Aug 4;7(8):e1002188. doi: 10.1371/journal.pgen.1002188 (PMC3150450; doi:10.1371/journal.pgen.1002188)
Supplement: Text S1 — Supporting Materials and Methods. (DOC) [file pgen.1002188.s005.doc]

**Text S1**

Ongoing Phenotypic and Genomic Changes in Experimental Coevolution of RNA Bacteriophage Qβ and *Escherichia coli*

Akiko Kashiwagi and Tetsuya Yomo

**Qβ and *E.coli* fitness assay.**

Cross-coculture experiments were conducted using Anc(C), M54(C), M163(C), and M165_2(C) as host populations and Anc(P), M54(P), M163(P), M165_2(P), and S94(P)_3 as phage populations as follows. Anc(C), M54(C), M163(C), and M165_2(P) were cultured in mM63gl at 37°C with shaking at 160 rpm overnight and transferred into fresh medium with dilution to initial OD_600_ = 0.03 (culture volume was 3 ml). After culture for 2 – 2.5 h, OD_600_ became 0.06 – 0.07. Dialyzed phage particles were then added and culture was continued at 37°C with shaking at 160 rpm. The free phage concentrations were analyzed at 0, 3.5, 7 h, and the end point (22 – 29.5 h later) by plaque assay according to the standard method [1]. Cell growth was monitored by measuring the OD_600_.

The time course experiments were conducted with Anc(C) infected with Anc(P), M54(C) infected with Anc(P) or M54(P), and M163(C) infected with Anc(P), M54(P), M163(P), or S94_3(P). *E. coli* was cultured in the same way as described in the cross-coculture experiment, with addition of dialyzed phage particles and culture at 37°C with shaking at 160 rpm. Sampling was performed at 0, 1, 2, 3, 4, 5, 7 – 8, and 23.5 – 33 h after infection. The culture samples were divided into two portions: one was centrifuged at 13,000 x *g* for 1 min at room temperature and the supernatant was used to measure the PFU as free phage density and the pellet was resuspended with mM63gl and diluted with LB medium as soon as possible, kept on ice until plaque assay and used to measure the plaque forming units (pellet-PFU) as infected cell density, while the other was diluted with buffer A(62 mM K_2_HPO_4_, 39 mM KH_2_PO_4_, 15 mM ammonium sulfate) and spread on mM63gl agar medium and cultured at 37°C for 2 days to measure CFU as total cell density. As this agar medium was low in divalent cations, reinfection of the cells by Qβ phage during cultivation was repressed, and there was little cell lysis. Therefore, the CFU values represent the viable cell numbers.

**Estimation of the rate constant of Qβ phage adsorption to the host bacteria.**

Anc(C) or M163(C) was cultured in mM63gl at 37°C overnight and transferred with dilution to initial OD_600_ = 0.03 (*n* = 3). After culturing for 2.5 h, aliquots were used to measure the concentration of Anc(C) or M163(C) as CFU on mM63gl, and dialyzed Anc(P) or dialyzed M163(P) was added to the rest of the liquid culture. After 0, 5, 10, 15, 30, and 60 min, nonadsorbed phage particles were separated using 0.2 μm syringe filters (Minisart RC15 filters; Sartorius Stedim Biotech). The nonadsorbed phage concentration was measured by plaque assay according to the standard method. The adsorption rate constant was calculated according to the method described previously [2]. The cell densities of Anc(C) infected with Anc(P) and M163(P) were 2.1×10^7^ CFU/ml (OD_600_ = 0.066) and 1.9×10^7^ CFU/ml (OD_600_ = 0.065), respectively, and that of M163(C) infected with M163(C) was OD_600_ = 0.09.

**REFERENCES**

1. Carlson K (2005) Appendix: Working with Bacteriophages: Common Techniques and Methodological Approaches. In: Kutter E, Sulakvelidze A, editors. BACTERIOPHAGES Biology and Applications. Boca Raton: CRC Press. pp. 437-494.

2. Tsukada K, Okazaki M, Kita H, Inokuchi Y, Urabe I, et al. (2009) Quantitative analysis of the bacteriophage Qβ infection cycle. Biochim Biophys Acta 1790: 65-70.
